# Application of two novel anionic peroxidases from *Raphanus sativus* L. var *niger* roots in labeling antibodies and developing an enzyme-linked immunosorbent assay

**DOI:** 10.1016/j.heliyon.2024.e40894

**Published:** 2024-12-13

**Authors:** Hooman Askari, Ali Nabati, Aliasghar Rahimian, Mahdi Aminian

**Affiliations:** Department of Clinical Biochemistry, School of Medicine, Tehran University of Medical Sciences, Tehran, Iran

**Keywords:** Peroxidase, *Raphanus sativus* L. var *Niger*, Black radish, Bioconjugation, Immunoassays

## Abstract

HRP, or horseradish peroxidase, is a reporter enzyme with extensive use in biotechnological applications. We previously reported the purification and characterization of two anionic peroxidases from *Raphanus sativus* L. var *niger* (black radish) roots. Here, we evaluated the applicability of these two novel peroxidases as alternatives to traditional horseradish peroxidase (HRP). The two novel peroxidases (BRP-A and BRP-B) and HRP were conjugated to IgY polyclonal antibodies by chemical methods based on the use of sodium periodate and cyanuric chloride. Moreover, the applicability of BRP-A and BRP-B in immunoassays was investigated by comparing the signal generated by these novel peroxidases in ELISA with HRP conjugates. Additionally, the limit of detection (LOD) was calculated for BRP-A, BRP-B, and HRP conjugates. Finally, the thermal stability of peroxidase antibody conjugates at 37 °C and 4 °C was compared. The peroxidase antibody conjugates prepared by the periodate method generated a much stronger signal than those prepared by the cyanuric chloride method. The signal obtained by BRP-A and BRP-B conjugates was much lower compared to the commercial HRP enzyme. The limit of detection was found to be 385.71, 213.75, and 43.6 ng per well for BRP-A, BRP-B, and HRP conjugates prepared by the periodate method, respectively. However, for conjugates prepared by the cyanuric chloride method, the limit of detection could only be estimated for HRP since BRP-A and BRP-B had an extremely low signal-to-noise ratio. All peroxidase conjugates had comparable thermal stability at 37 °C and 4 °C.

## Highlights

• Two novel peroxidases purified from black radish were compared to traditional horseradish peroxidase.
• IgY antibodies against diphtheria toxoid were conjugated to peroxidases using two chemical conjugation methods.
• IgY-peroxidase conjugates were used to develop an enzyme-linked immunosorbent assay.
• The conjugates obtained by the periodate method yielded a much stronger signal in ELISA.
• Signal obtained by BRP-A and BRP-B conjugates was much lower compared to the commercial HRP enzyme.

## Graphical abstract

**Figure:**
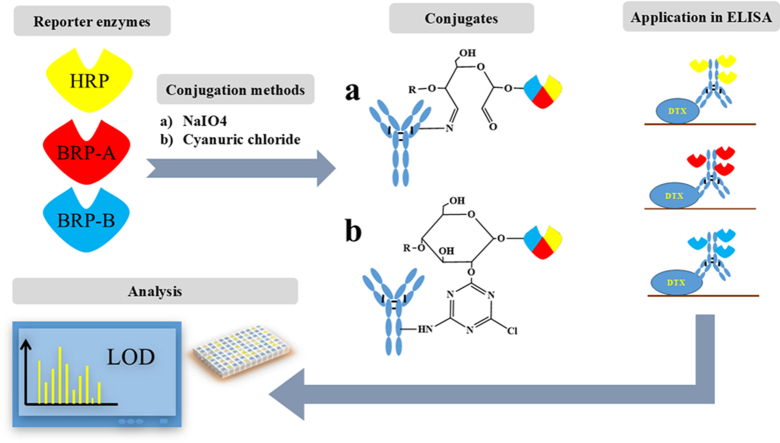

## 1 Introduction

The term peroxidase (1.11.1.7) describes a large family of oxidoreductase enzymes that utilize a variety of peroxides to catalyze the oxidation of organic and inorganic substrates. Peroxidases play various biological roles in different organisms [1]. Plant peroxidases are especially of interest in biological applications due to their ability to oxidize aromatic compounds. As dyes generally possess an aromatic structure, the ability of peroxidases to act on such compounds has enabled researchers to develop an exceptionally large set of chromogenic substrates to perform a wide range of qualitative and quantitative measurements. Peroxidase chromogenic substrates can be converted into soluble or precipitating products (and sometimes both) by the action of the enzyme at the expense of hydrogen peroxide. Substrates with the soluble product (such as 4-aminoantipyrine and o-phenylene diamine) are commonly used for quantitative measurements such as ELISA, while precipitating substrates (like 3,3′-diaminobenzidine) are used in immunohistochemical and blotting techniques. A brief list of soluble and precipitating peroxidase substrates routinely used by researchers is provided in Supplementary Table S1. The most well-known source of peroxidase for biotechnological applications is horseradish, or *Armoracia rusticana*, which can be cultivated in cold climates [2,3]. Horseradish peroxidase (HRP) has widespread application in immunodiagnostic products as a reporter enzyme [4]. While other reporter enzymes such as β-galactosidase and alkaline phosphatase can be used for this application, its high stability and small size have made HRP far more popular than any other enzyme [5]. Workers have also examined other sources as alternatives to horseradish, as the use of HRP cannot be justified economically in all situations. Soybean is one of the most successful cases of peroxidase alternative sources. Soybean peroxidase (SBP) shows great stability in a wide range of pH and temperature and has been employed with success as a substitute for HRP in many applications [1,6]. Recently, studies have investigated the efficacy of SBP in immunoassays and even managed to achieve a limit of detection lower than that of HRP conjugates [7,8]. Peroxidases from other sources have also been investigated and employed in immunoassays [[9], [10], [11]]. Besides the source of the enzyme, the conjugation method should be taken into account in an investigation of reporter enzymes. There are many different chemical conjugation methods based on functional groups such as amine, carboxyl, thiol, and hydroxyl on the surface of the protein. HRP is traditionally coupled to free amine groups of antibodies through its carbohydrate moiety by the periodate oxidation method. What initially prompted Nakane et al. to develop this approach was the low efficacy of the glutaraldehyde method of coupling HRP to antibody. Glutaraldehyde is a homo-bifunctional cross-linker that acts on amine groups, and it was extensively used in the field of bioconjugation. Nakane et al. believed that the modification of primary amines on HRP with allylisothiocyanate, an organosulfur compound found abundantly in *Armoracia rusticana*, was the reason for the glutaraldehyde method's ineffectiveness in coupling HRP to antibody [12]. This shows that varying results might be obtained with different peroxidases depending on the nature of the enzyme, its source, and the conjugation method employed. Therefore, the examination and optimization of conjugation methods should be considered crucial in investigating alternative reporter enzymes. Cyanuric chloride has been proposed as another method for coupling HRP to antibodies. The method can be considered rather similar to periodate oxidation, as cyanuric chloride also generates a covalent bond between the hydroxyl groups on the carbohydrate moiety of HRP and the amine groups on the surface of the antibody.

We previously reported the purification of two anionic peroxidase enzymes from black radish roots (isoenzyme A and B, which will be called BRP-A and BRP-B henceforth) with a high degree of chemical and thermal stability [13]. Here, we aimed to examine the applicability of these two enzymes in labeling antibodies compared with traditional HRP. Peroxidase enzymes BRP-A, BRP-B, and HRP were conjugated to purified IgY polyclonal antibodies using periodate and cyanuric chloride methods. The signal generated from these conjugates was measured and compared using ELISA. Moreover, the limit of detection (LOD) was determined for conjugates obtained by both methods.

## 2 Materials and methods

### 2.1 IgY polyclonal antibody

IgY polyclonal antibody was generated against diphtheria toxoid (DTx) and purified by polyethylene glycol precipitation in a previous report [14]. This antibody was used in the preparation of all peroxidase antibody conjugates without further purification, and hereinafter is referred to as the IgY polyclonal antibody.

### 2.2 Optimization of periodate concentration for conjugation of novel peroxidases to IgY polyclonal antibody

As the stability of the two novel isoenzymes (BRP-A and BRP-B) towards oxidation by periodate has not been investigated previously, the enzymes BRP-A, BRP-B, and HRP were subjected to different concentrations of this oxidizing agent.

HRP enzyme was obtained from Biobasic, Canada. BRP-A and BRP-B were extracted and purified from black radish roots in our previous study; however, here, we did not use the concanavalin A affinity column for the purification of BRP-A and BRP-B since it leads to a huge reduction in yield. These two enzymes were previously characterized, and their kinetic parameters are presented in Supplementary Table S2 and also in the original work [13]. The total protein concentration of peroxidases was determined by the Bradford method as described elsewhere, using bovine serum albumin (BSA) as standard [15]. All three peroxidases were treated with 1-fluoro-2,4-dinitrobenzene (FDNB) to minimize self-coupling. Peroxidases were diluted to a concentration of 5 mg/mL, and then FDNB stock solution (10 mg/mL, in absolute ethanol) was added (one-tenth of the total volume of solution). The solution was incubated for 2 h at RT and then dialyzed against 10 mM carbonate, pH 9.5, as described by Nakane et al. [12]. Subsequently, these three peroxidases were diluted to a protein concentration of 1 mg/mL in 100 mM carbonate buffer, pH 8.1, and each was distributed into 22 microtubes with a volume of 300 μL. Periodate (Biobasic, Canada) was dissolved in deionized water and serially diluted to obtain concentrations ranging from 1 to 512 mM (ten concentrations in total). Immediately afterward, 300 μL of each periodate dilution were added to the microtubes containing diluted peroxidases in duplicate, and 300 μL of deionized water were also added to a pair of these microtubes as control. The reaction was allowed to proceed in the dark at room temperature for exactly 2 h, and then it was terminated by adding 60 μL of glycerol to each microtube. A 60-μL sample was taken from each tube and was used for the determination of enzymatic activity. Subsequently, the IgY polyclonal antibody was diluted to a concentration of 0.5 mg/mL in 100 mM carbonate buffer, pH 9.2, and added to each tube with a volume of 600 μL. The microtubes were incubated at RT overnight, and a 20-μL sample was taken for enzyme assay. Afterward, sodium borohydride was dissolved in 0.1 mM sodium hydroxide solution at a concentration of 5 mg/mL and was immediately added to the microtubes with a volume of 228 μL. The reaction was allowed to continue at RT for 2 h, and then the enzyme assay was performed on samples from this step using phenol and 4-aminoantipyrine as substrates, as described in our previous study [13]. The loss of total enzymatic activity upon activation, conjugation to IgY polyclonal antibody, and reduction with sodium borohydride was investigated by comparing the relative activity of peroxidases with their corresponding controls.

In order to find the optimum periodate concentration for conjugation of each peroxidase enzyme to the IgY polyclonal antibody, direct ELISA was performed. The goal of this assay was to determine the periodate concentrations that yield conjugates capable of generating the highest signal. Diphtheria toxoid (DTx) was diluted with 50 mM carbonate buffer, pH 9.4, to obtain the final concentration of 10 μg/mL, and 50 μL were loaded in 90 wells. The microplate was incubated overnight at 4 °C, and then the wells were washed three times with 250 μL of PBST (phosphate-buffered saline + 0.1 % V/V Tween 20). Afterward, blocking was done using 5 % skim milk dissolved in PBST. Again, the microplate was washed three times with PBST. All peroxidase-antibody conjugates were diluted to obtain a final concentration corresponding to 100 μg/mL of IgY polyclonal antibody using 2 % skim milk dissolved in PBST. The conjugates were applied in a volume of 100 μL in triplicate, and the microplate was incubated at 37 °C for 2 h. Unbounded conjugates were removed by three consequent washes using PBST. Lastly, 100 μL of TMB substrate solution (containing 2 mM TMB and 2 mM H_2_O_2_ in 100 mM citrate, pH 5) were added to each well, and the plate was incubated in the dark at RT. The absorbance was read at 375 nm after 10 min. The reaction was terminated by the addition of 100 μL of 2M HCl after 20 min, and the absorbance was measured at 450 nm. The signal intensity was reported as mean value ± RSE (relative standard error), and graphs were generated using GraphPad Prism 9.0.0 (GraphPad Software Inc., USA).

### 2.3 Conjugation of BRP-A, BRP-B, and HRP to IgY polyclonal antibody using periodate and cyanuric chloride methods

The conjugation of peroxidases to the IgY polyclonal antibody was performed by two chemical methods based on the use of periodate and cyanuric chloride.

First, free amine groups of peroxidase enzymes were blocked using 1-fluoro-2,4-dinitrobenzene (FDNB) according to the same procedure described in the previous section. The UV–visible spectra in the range of 200–460 nm were recorded before and after the treatment of each enzyme with FDNB. GraphPad Prism 9.0.0 was used to generate the graphs.

The periodate coupling was performed according to the protocol described by Tijssen et al. [16]. BRP-A, BRP-B, and HRP were diluted to protein concentrations of 2, 4, and 1 mg/mL in 100 mM carbonate buffer, pH 8.1, in 1 mL volumes, and were mixed with an equal volume of 8 mM periodate dissolved in deionized water. The reaction was allowed to proceed at RT in the dark for 2 h. Subsequently, 416.6 mg of dry Sephadex G-25 and 500 μL of IgY polyclonal antibody with a protein concentration of 2 mg/mL in 500 mM carbonate, pH 9.2, were added to the reaction mixture. The suspension was mixed and incubated overnight at RT. The supernatant solution was taken (1 mL), and then the resin was washed with 2 mL of deionized water to recover some of the remaining trapped conjugates. Subsequently, the conjugates (with an approximate volume of 3 mL) were reduced through the addition of 5 mg/mL sodium borohydride solution at RT in two steps: first, 150 μL with 30 min incubation, then 450 μL with 1 h incubation. The conjugates were kept at 4 °C for further analysis.

Conjugation was also done according to a more recent method described by Abuknesha et al. with some slight modifications [17,18]. Cyanuric chloride (Sigma-Aldrich, Germany) was dissolved in acetonitrile at 140 mg/mL concentration. BRP-A, BRP-B, and HRP were diluted to a protein concentration of 2, 4, and 1 mg/mL in 50 mM carbonate buffer (activation buffer), pH 9.4, in volumes of 4 mL for BRP-A and BRP-B and 3 mL for HRP, in glass tubes, and cooled down on ice for 30 min. Subsequently, 50 μL of 140 mg/mL cyanuric chloride solution were carefully added to each tube and gently mixed. The tubes were incubated on ice for 4 h and then centrifuged (1000g, 15 min). Subsequently, the supernatant solutions were separated from unsolved cyanuric chloride, and dialysis was done against 1 L of the same activation buffer using 10 kDa cut-off dialysis bags for 4 h at 4 °C. For HRP, the dialyzed enzyme was poured into three separate microtubes in equal volumes of 1 mL. IgY polyclonal antibody dissolved in 50 mM carbonate, pH 9.4, was added to each tube in volumes of 1 mL in concentrations of 100, 500, and 1000 μg/mL. The same procedure was done for BRP-A and BRP-B with 100, 500, 1000, and 1500 μg/mL IgY concentrations. The incubation was done for 48 h at 37 °C, and then further analysis was done.

### 2.4 Determination of free amine groups in conjugates using the OPA method

The free amine content of conjugates was determined according to the method described by Goodno et al. using ortho-phthalaldehyde (OPA) [19]. OPA stock solution was prepared by dissolving 40 mg of OPA (Merck, Germany) in absolute ethanol. A fresh reagent was prepared immediately before the assay by mixing 2 mL of OPA stock with 50 mL of 100 mM sodium tetraborate, pH 9.5, 5 mL of 20 % W/W SDS, and 0.2 mL of 2-mercaptoethanol. Protein samples were added with a volume of 50 μL to 3 mL of fresh reagent in a 3 mL fluorescence quartz cuvette, and the fluorescence intensity was measured after 2 min using an FP-6200 spectrofluorometer (Jasco, Japan) at 25 °C. The excitation was done at 340 nm, and the emission was measured at 455 nm. IgY polyclonal antibodies with concentrations of 50, 250, and 500 μg/mL were used as standards. The emission intensity of the conjugates was measured and compared to the IgY standard (GraphPad Prism 9.0.0).

### 2.5 Comparison of the signal intensity of the conjugates obtained from periodate and cyanuric chloride methods by direct ELISA

In order to compare the efficacy of periodate and cyanuric chloride conjugation methods of labeling antibodies, a direct ELISA method was used. Diphtheria toxoid was immobilized on the surface of two Nunc Polysorp plates by loading 50 μL of antigen with a concentration of 10 μg/mL in 50 mM carbonate, pH 9.6, (coating buffer) following incubation at 4 °C overnight to obtain 500 ng per well antigen concentration. The wells were washed three times with 250 μL of PBST (phosphate-buffered saline + 0.1 % V/V Tween 20) the following day. Subsequently, the wells were blocked using 250 μL of 5 % skim milk in PBST for 2 h of incubation at 37 °C. Again, washing was done three times with PBST. Conjugates were then applied with three IgY concentrations of 10, 20, and 40 μg/mL in a volume of 100 μL diluted with 3 % skim milk in PBST. After 2 h of incubation at 37 °C, washing was done three times to remove the unbounded conjugates. Following that, 100 μL of TMB substrate solution (containing 2 mM TMB and 2 mM H_2_O_2_ in 100 mM citrate, pH 5) were added to each well, and the plate was incubated in the dark for 20 min at RT. The reaction was terminated by adding 100 μL of 2M HCl, and the absorbance was measured at 450 nm. The data was plotted using GraphPad Prism 9.0.0.

### 2.6 Determination of the limit of detection (LOD) for conjugates

The limit of detection (LOD) was measured to further investigate the efficacy of the two conjugation methods for HRP and the two novel peroxidases, BRP-A and BRP-B. Several approaches exist for determining LOD. In this study, LOD was defined as three standard deviations of negative controls divided by the slope of the calibration curve [20]. 20 blank replicates (negative controls) were used for each conjugate, and the calibration curve was generated with triplicates of diphtheria toxoid concentrations ranging from 23 to 3000 ng per well.

$$LOD=\frac{3*SD\;of\;blanks}{Slope\;of\;standard\;curve}$$

Diphtheria toxoid was diluted in coating buffer to obtain a final protein concentration of 10 μg/mL, and 100 μL of this antigen were loaded in three wells. The antigen was serially diluted three times in the plate using coating buffer to reach the final concentration of 1.25 μg/mL, which corresponds to 62.5 ng/well antigen concentration. The plate was incubated overnight at RT. Washing was done three times the following day using 250 μL of PBST, and then the plate was blocked with 250 μL of 5 % skim milk in PBST and 2 h of incubation at 37 °C. The plate was washed three times again using PBST. Subsequently, conjugates were added with a constant IgY concentration, which was previously determined with a checkered board experiment, in a volume of 100 μL to each well. After the plate was incubated for 2 h at 37 °C, the washing was done again using PBST. Finally, 100 μL of TMB substrate solution were added to each well, and the plate was incubated in the dark at RT for 20 min. The reaction was stopped by the addition of 100 μL of 2M HCl, and the absorbance was measured at 450 nm.

### 2.7 Thermal stability of conjugates

In order to examine their thermal stability, peroxidase-antibody conjugates obtained by the periodate method were incubated at 4 °C and 37 °C. The conjugates were diluted to a concentration equivalent to 100 μg of IgY polyclonal antibody per mL in PBS, and several aliquots were made. The aliquots were kept at 4 °C and 37 °C. An aliquot was taken from each conjugate on days 10, 20, 30, 40, and 60 and was stored at −20 °C (with the addition of glycerol as a cryoprotectant to a final concentration of 50 %) until ELISA was performed. Diphtheria toxoid was coated on a Nunc Polysorp plate with a concentration of 500 ng per well, and conjugates were applied with an antibody concentration of 40 μg/mL for BRP-A and BRP-B and 10 μg/mL for HRP conjugate in a volume of 50 μL in duplicate. A separate negative control well was designated for every conjugate obtained on the specified days. The intensity of the signal generated by each of the conjugates incubated at these two temperatures was measured in the same way we described earlier for comparing conjugation methods. GraphPad Prism 9.0.0 was used to illustrate the data.

## 3 Results and discussion

### 3.1 Periodate effect on peroxidase enzymatic activity

The residual activity of peroxidases after exposure to periodate is summarized in Supplementary Fig. S3. Three peroxidases (BRP-A, BRP-B, and HRP) were subjected to periodate, a common oxidizing reagent used in bioconjugation, in concentrations ranging from 0.5 to 256 mM to determine their stability towards this reagent. The peroxidases were treated with FDNB beforehand to minimize self-coupling and diluted to a concentration of 1 mg/mL. The activity of peroxidases subjected to periodate was compared to the FDNB-treated peroxidases and reported as relative activity ± relative standard error (RSE). Control samples (n = 9) had an RSE of 4.2, 15.1, and 10.0 for BRP-A, BRP-B, and HRP, respectively. FDNB treatment reduces the specific activity of peroxidases from 166.7, 64.8, and 366.1 U/mg to 146.0, 53.6, and 269.2 U/mg for BRP-A, BRP-B, and HRP, respectively. As can be seen in Supplementary Fig. S3, BRP-A and BRP-B showed slightly higher stability as compared to HRP in the periodate coupling method. However, it should be mentioned that what we measured in our assay was the total enzymatic activity of both conjugated and unconjugated HRP. Therefore, a high enzymatic activity observed in this experiment does not necessarily mean a high signal from the conjugates, as the conjugate might lack enzymatic activity or the ability to bind to the antigen. A more accurate estimation might be obtained by purifying coupled antibodies and then performing the enzyme assay, or better, by implementing an ELISA method.

**Fig. 1:**
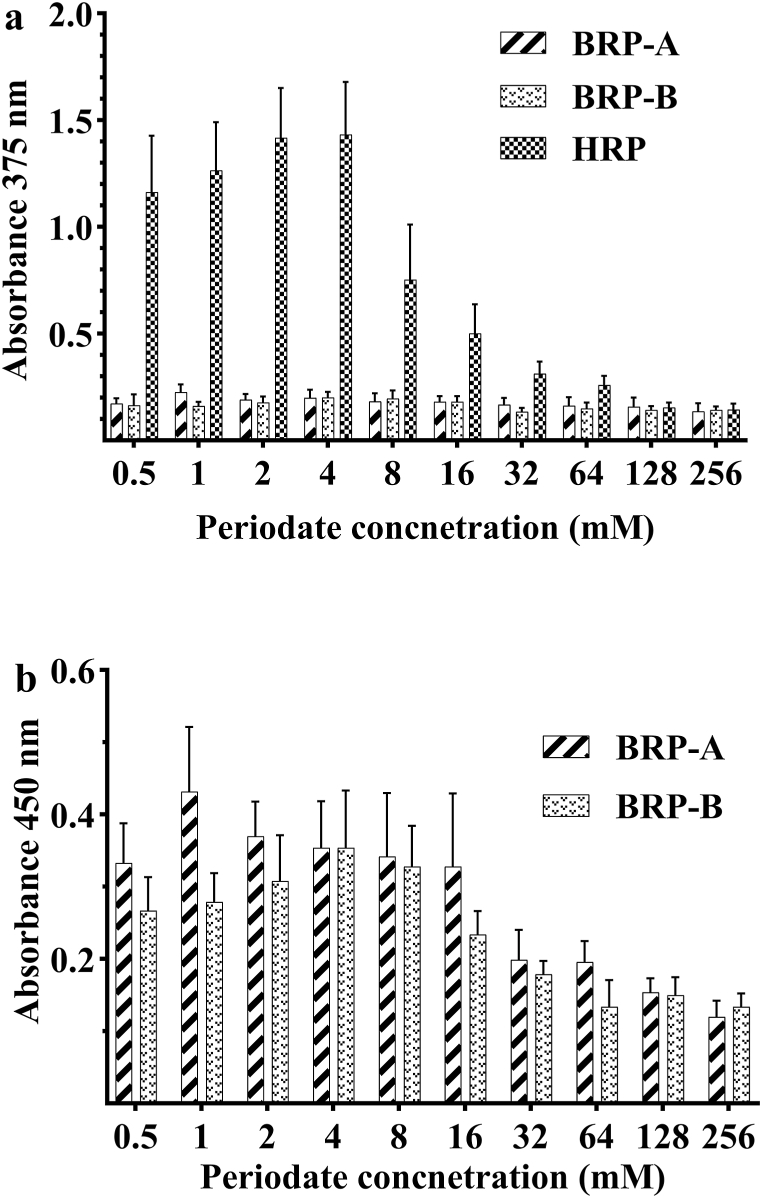
Comparison of the signal generated from conjugates prepared by various concentrations of periodate. (a) The absorbance was measured after 10 min at 375 nm. (B) The absorbance was measured after 20 min at 450 nm.

The data obtained from direct ELISA is presented in Fig. 1. Since HRP-IgY conjugate gave a significantly higher signal than BRP-A and BRP-B conjugates, the absorbance was measured at 375 nm after 10 min to avoid a non-linearity between product concentration and absorbance. The highest optical density was observed at 4 mM periodate concentrations for BRP-B and HRP, but at 1 mM concentrations for BRP-A. In addition, the signal obtained from BRP-A and BRP-B was comparable in the range of 0.5–16 mM periodate concentration. However, HRP conjugate seems to lose a large portion of its enzymatic activity at concentrations above 4 mM. Based on this data, we decided to perform the conjugation experiment at 4 mM concentrations for all peroxidases.

### 3.2 Conjugation of BRP-A, BRP-B, and HRP to IgY polyclonal antibodies generated against DTx using periodate and cyanuric chloride methods

Two novel peroxidase isoenzymes (BRP-A and BRP-B) and HRP were conjugated to IgY polyclonal antibodies generated against DTx using two previously described methods based on the use of periodate and cyanuric chloride.

Conjugation by periodate oxidation was done according to the procedure described by Tijssen et al., in 1984. The free amine groups of the enzyme were blocked using the FDNB treatment to avoid self-coupling. The molar ratio of enzyme to antibody differed for each peroxidase enzyme and was approximately 3, 7, and 4 for BRP-A, BRP-B, and HRP, respectively.

Conjugation of the peroxidase to the antibody using cyanuric chloride was done according to a modified version of what was first described by Abuknesha in 2005. The peroxidases were treated with FDNB beforehand to avoid self-coupling. Four different molar ratios of peroxidase to antibody were employed for BRP-A and BRP-B, ranging from 2:1 to 35:1 and 5:1 to 74:1, respectively. HRP was also conjugated to the IgY antibody in three different molar ratios, ranging from 4:1 to 41:1.

The loss of total enzymatic activity during FDNB treatment and conjugation for each peroxidase is summarized in Supplementary Fig. S4. In addition, the change in UV–visible spectra of each peroxidase can be seen in Supplementary Fig. S5. The steep increase in absorbance around 360λ for BRP-A and BRP-B shows that a high number of free amine groups are present on the surface of these enzymes, as opposed to HRP, which bears six lysine residues on its surface. It has been suggested that amine groups on the surface of HRP are mostly modified by allylisothiocyanate, which is produced in large quantities by *Armoracia rusticana*, and thus cannot participate in coupling reactions [12,21]. However, while the highly pungent odor of black radish extract might indicate the presence of at least some kind of glucosinolate metabolites, this does not seem to be the case with BRP-A and BRP-B, as FDNB reacts readily with both of these isoenzymes.

### 3.3 Determination of free amine groups in conjugates using the ortho-phthaldehyde method

The free amine content of conjugates was estimated by a fluorometric method using ortho-phthaldehyde (OPA), as Goodno et al. described. A standard curve was generated using various concentrations of IgY polyclonal antibody, and using a simple linear regression analysis, a formula was generated to estimate the amount of free amine groups for each conjugate (Supplementary Fig. S6). As the enzymes used for the conjugation reaction are treated with FDNB beforehand, it is expected that the measured fluorescence intensity is due to the presence of free-amine groups on the surface of the IgY polyclonal antibody. The fluorescence intensity obtained from conjugates was compared to the standard curve, and the results are summarized in Supplementary Fig. S7. As it can be seen, increasing the ratio of enzyme to IgY polyclonal antibody did not significantly alter the free-amine content of the conjugates obtained by the cyanuric chloride method. It seems that most amine groups on the IgY polyclonal antibody have reacted with the enzymes, and the measured fluorescence activities might just be the result of reactions with non-reactive sites that play no role in the coupling of the enzyme.

### 3.4 Comparison of the signal intensity of the conjugates obtained from periodate and cyanuric chloride methods by direct ELISA

**Fig. 2:**
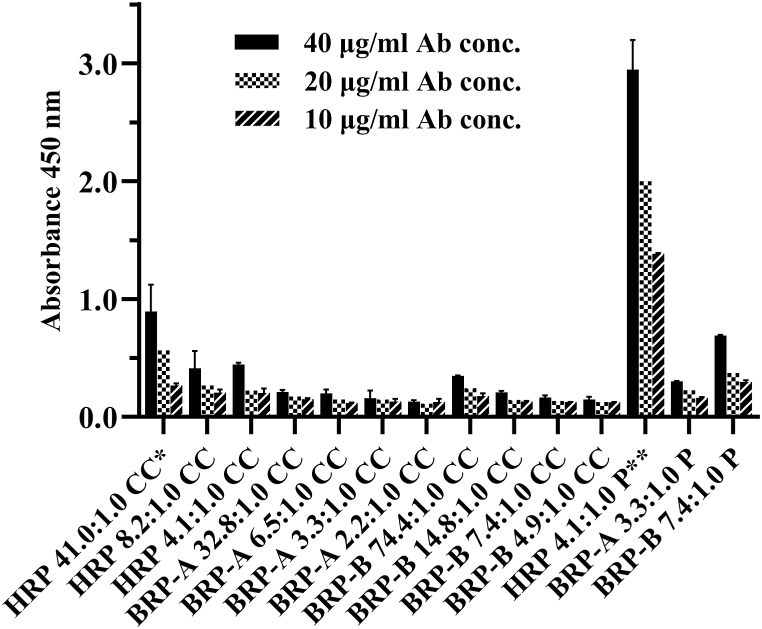
Comparison of the signal generated in ELISA by each conjugate at 40, 20, and 10 μg/mL antibody concentrations. CC and P indicate the cyanuric chloride and periodate methods, respectively. HRP-antibody conjugate prepared by the periodate method gave the highest signal.

In order to evaluate the ability of peroxidase antibody conjugates to generate signals, a direct ELISA method was employed. The antigen (DTx) was coated with a constant concentration of 500 ng per well. The conjugates were applied in three different concentrations, namely 40, 20, and 10 μg/mL, in a volume of 100 μL. The results are represented in Fig. 2. The highest signal was obtained from HRP-Ab conjugates prepared by the periodate method. As shown, increasing the molar ratio of peroxidase to antibody increases the signal generated by all three peroxidases. In addition, it was observed that the periodate method has a higher efficacy, as conjugates prepared by this method produced the highest signal.

### 3.5 Determination of the limit of detection (LOD) for conjugates

**Fig. 3:**
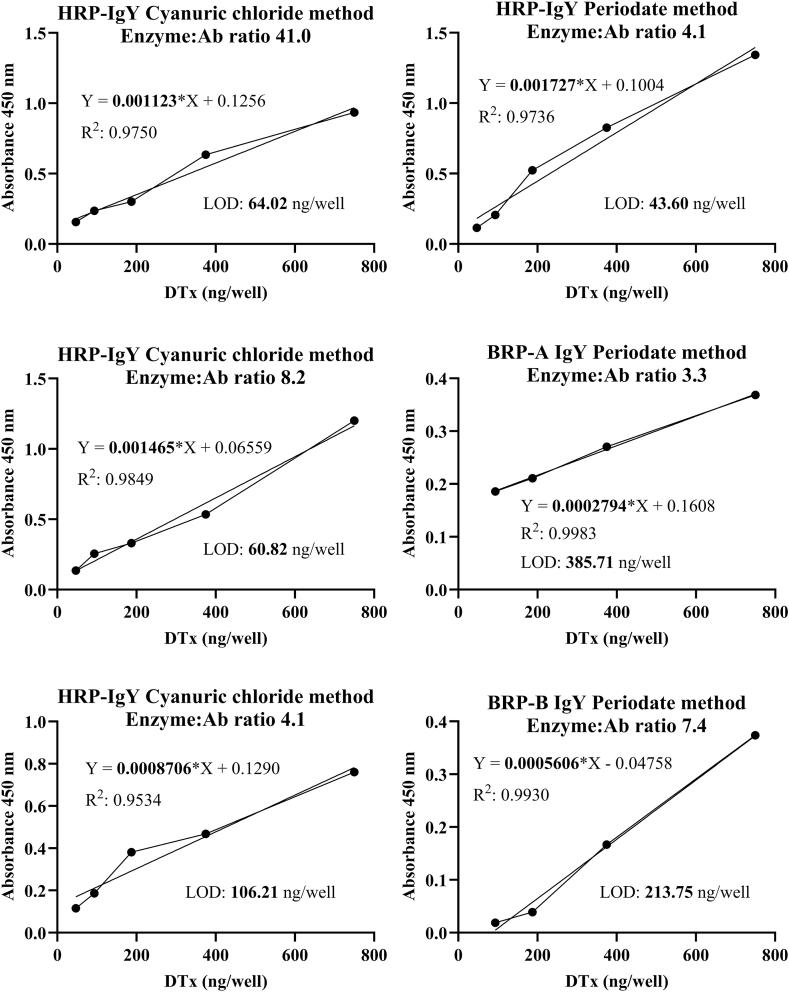
The standard curves generated using various antigen concentrations (DTx) and different conjugates. The slope of the calibration curve was estimated by fitting a simple linear regression model.

The limit of detection (LOD) was calculated for each conjugate to evaluate their suitability further and examine the suitability of the two conjugation methods for each peroxidase. The standard curves were generated for each conjugate and the standard deviation of 20 blanks was measured to estimate the limit of detection through the aforementioned formula. The results are presented in Fig. 3 and Supplementary Table S8. As can be seen, the lowest limit of detection was achieved using commercial HRP. The LOD could not be calculated for BRP-A and BRP-B conjugates obtained by the cyanuric chloride method since the signal was too low and a standard curve could not be generated. Overall, this experiment confirmed our findings in the previous section by comparing the signal generated by each conjugate. One factor responsible for the weak signal generated by BRP-A and BRP-B conjugates might be the low specific activity obtained by the purification process. The HRP had a specific activity of 360 U/mg, which is more than three times higher than that of BRP-A and BRP-B. However, it seems unlikely that this lower specific activity alone is responsible for the extremely poor signal generated by BRP-A and BRP-B conjugates. Another justification might be the large size of anionic peroxidases purified from black radish, which is 110 and 97 KDa for BRP-A and BRP-B, respectively. As HRP has a molecular weight of around 44 KDa, a higher number of enzymes could theoretically be able to bind to a single molecule of IgY antibody due to a lower steric hindrance. Also, it might be possible that black radish peroxidases lose their activity after the conjugation to antibody, and the total activity observed is owing to the presence of the uncoupled enzyme; however, this seems rather unlikely as the results of the orto-phtaldehyde assay confirmed the extensive degree of cross-linking.

### 3.6 Thermal stability of conjugates

**Fig. 4:**
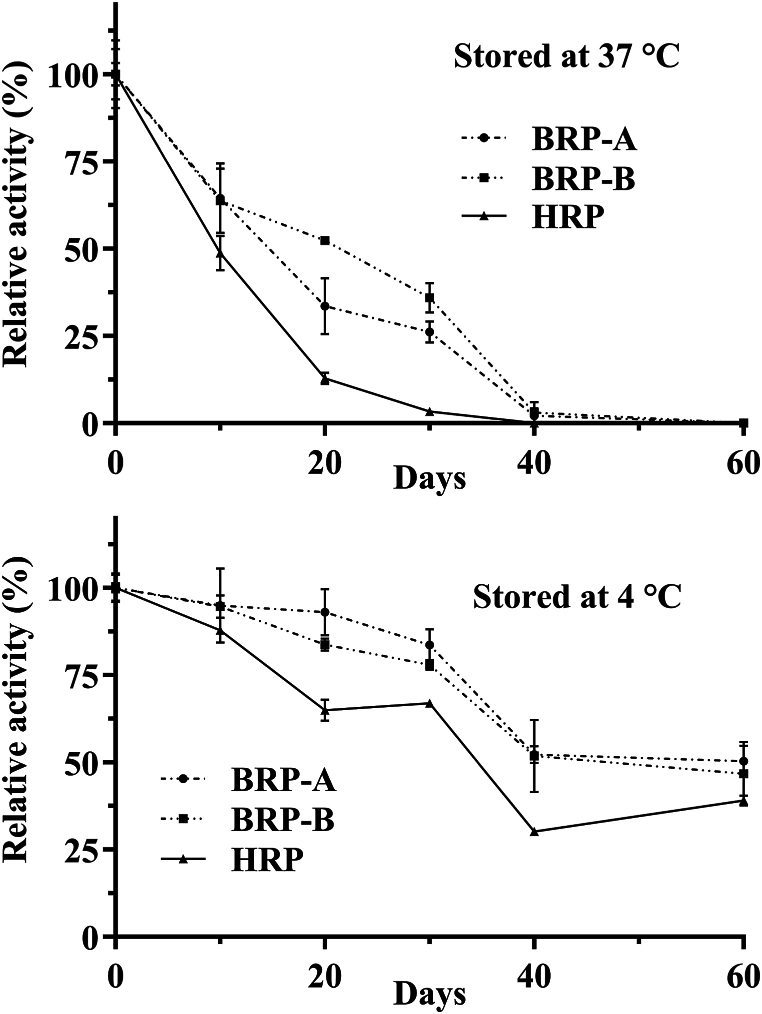
The thermal stability of BRP-A, BRP-B, and HRP conjugates measured using ELISA.

The thermal stability of BRP-A, BRP-B, and HRP antibody conjugates was compared (Fig. 4). Two of the most important factors that should be taken into account in an investigation of protein stability are protein concentration and the composition of the storage buffer. Here, the aliquots contained 100 μg/mL of IgY polyclonal antibody and its corresponding peroxidase in molar ratios of 4.1, 3.3, and 7.4 for HRP, BRP-A, and BRP-B, respectively. In addition, the enzymes were diluted using PBS buffer. As expected, all conjugates lose their activity much faster at 37 °C. BRP-A and BRP-B conjugates seem to be slightly more stable; however, it should be considered that these conjugates contain a higher total protein content compared to HRP due to the high molecular weight of the novel peroxidase and the molar ratio of enzyme to antibody employed. Therefore, higher thermal stability cannot be inferred for BRP-A and BRP-B conjugates.

## 4 Conclusion

BRP-A and BRP-B, two novel peroxidases from black radish roots, were conjugated to an IgY polyclonal antibody and were subsequently used for the detection of diphtheria toxoid (DTx). The cyanuric chloride and periodate chemical conjugation methods resulted in a lower loss of total enzymatic activity for BRP-A and BRP-B compared to HRP; however, the novel peroxidases yielded a significantly lower signal in ELISA compared to commercial HRP. Additionally, we determined the limit of detection for BRP-A, BRP-B, and HRP conjugates prepared by the periodate method to be 385.71, 213.75, and 43.6 ng/well, respectively. The LOD could not be determined for BRP-A and BRP-B conjugates prepared by the cyanuric chloride method. All conjugates showed satisfactory thermal stability. Overall, HRP seems to be a much more suitable option for labeling antibodies and performing immunoassays. It should be mentioned that the specific activities of the enzymes that we managed to purify from black radish roots were much lower compared to the commercial HRP preparations. In addition, the use of linkers and spacers, which are widely used today, could improve the results. Newer conjugation methods are also known to create superior enzyme-antibody conjugates. Amine-to-sulfhydryl heterobifunctional crosslinkers that react with the thiol groups of the antibody and amine groups on the surface of the enzyme are among the most popular choices for developing enzyme-linked immunosorbent assays. In this study, however, we only investigated two chemical conjugation methods that followed nearly the same principle.

Isolating peroxidases from sources other than horseradish seems to be a promising approach for finding cost-efficient sources of a label enzyme in parts of the world where cultivation of the plant is not possible. Nonetheless, employing alternative peroxidases would not be an easy task, as most of the current conjugation methods and assays are based on the traditional HRP. In our experience, HRP is a superior reporter enzyme for developing immunoassays by far. For applications such as waste water treatment, on the other hand, black radish might provide a cheap source of crude peroxidase enzyme with considerable thermal and chemical stability.

### Funding

This work has received partial financial support from 10.13039/501100004484Tehran University of Medical Sciences and Health Services (grant number 46714).

## CRediT authorship contribution statement

**Hooman Askari:** Writing – review & editing, Writing – original draft, Project administration, Methodology, Investigation, Conceptualization. **Ali Nabati:** Writing – review & editing, Investigation, Conceptualization. **Aliasghar Rahimian:** Writing – review & editing, Supervision, Conceptualization. **Mahdi Aminian:** Writing – review & editing, Supervision, Project administration, Funding acquisition, Conceptualization.

## Consent to participate

Not applicable.

## Consent to publish

Not applicable.

## Ethics approval

There was no involvement of human or animal subjects in this study except for the IgY polyclonal antibody used, which was obtained from a previous project done with the approval of Ethics Committee of the Tehran University of Medical Sciences (No 24271–284541).

## Availability of data and materials

The authors declare that the data supporting the findings of this study are available within the paper. Other relevant data might be provided upon request to the corresponding author.

## Declaration of competing interest

The authors declare that they have no known competing financial interests or personal relationships that could have appeared to influence the work reported in this paper.

## Figures and Tables

**Figure:**
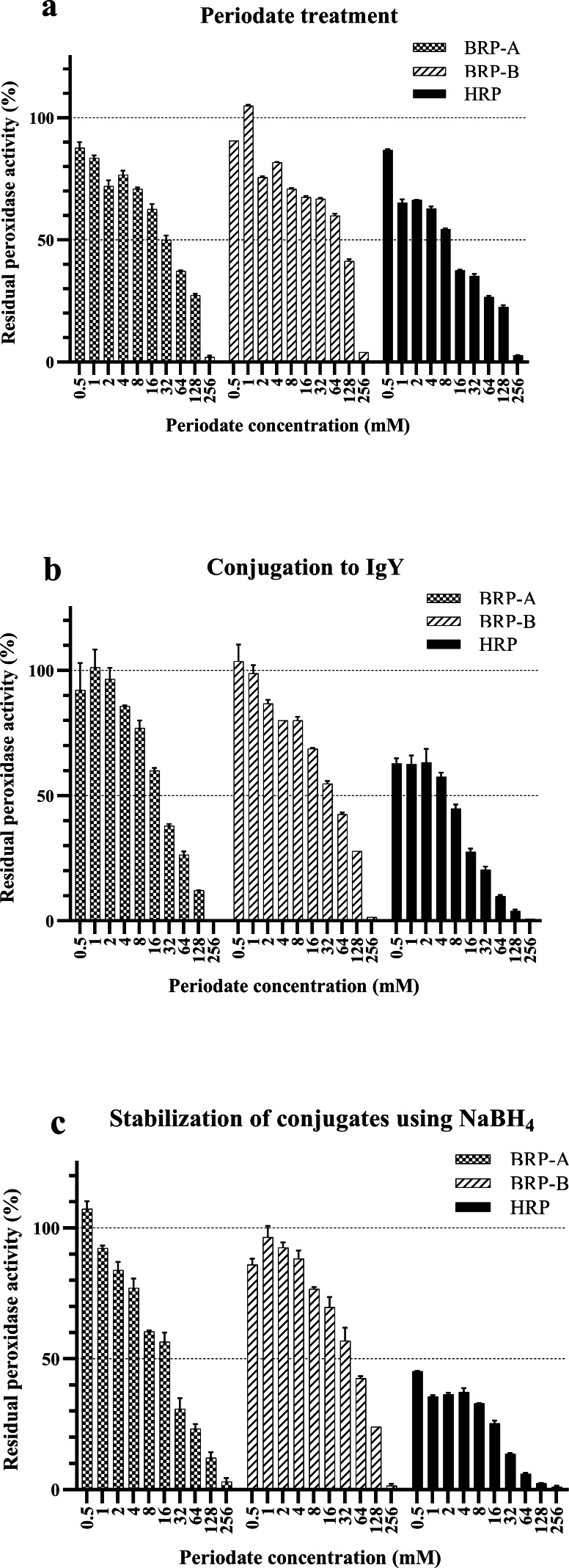
Supplementary Fig. S3. Residual activity of peroxidases (%) after exposure to various concentrations of periodate during the activation of the enzyme (a), conjugation to IgY (b), and reduction with NaBH_4_ (c).

**Figure:**
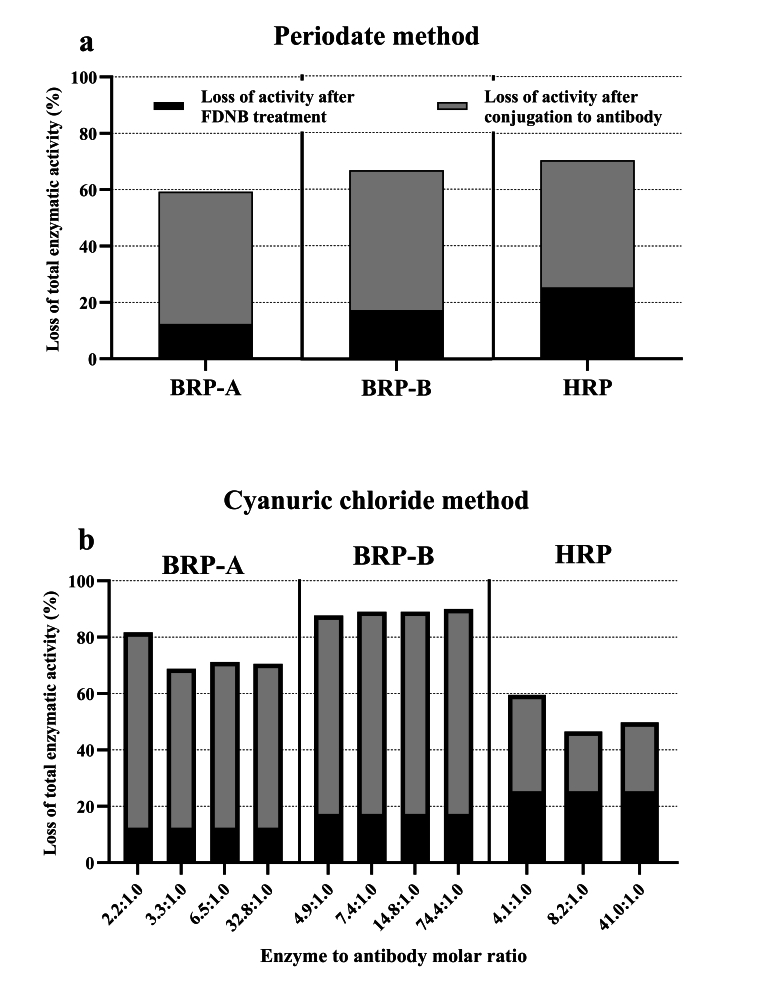
Supplementary Fig. S4. The loss of total enzymatic activity during periodate (a) and cyanuric chloride (b) conjugation methods for HRP, BRP-A, and BRP-B.

**Figure:**
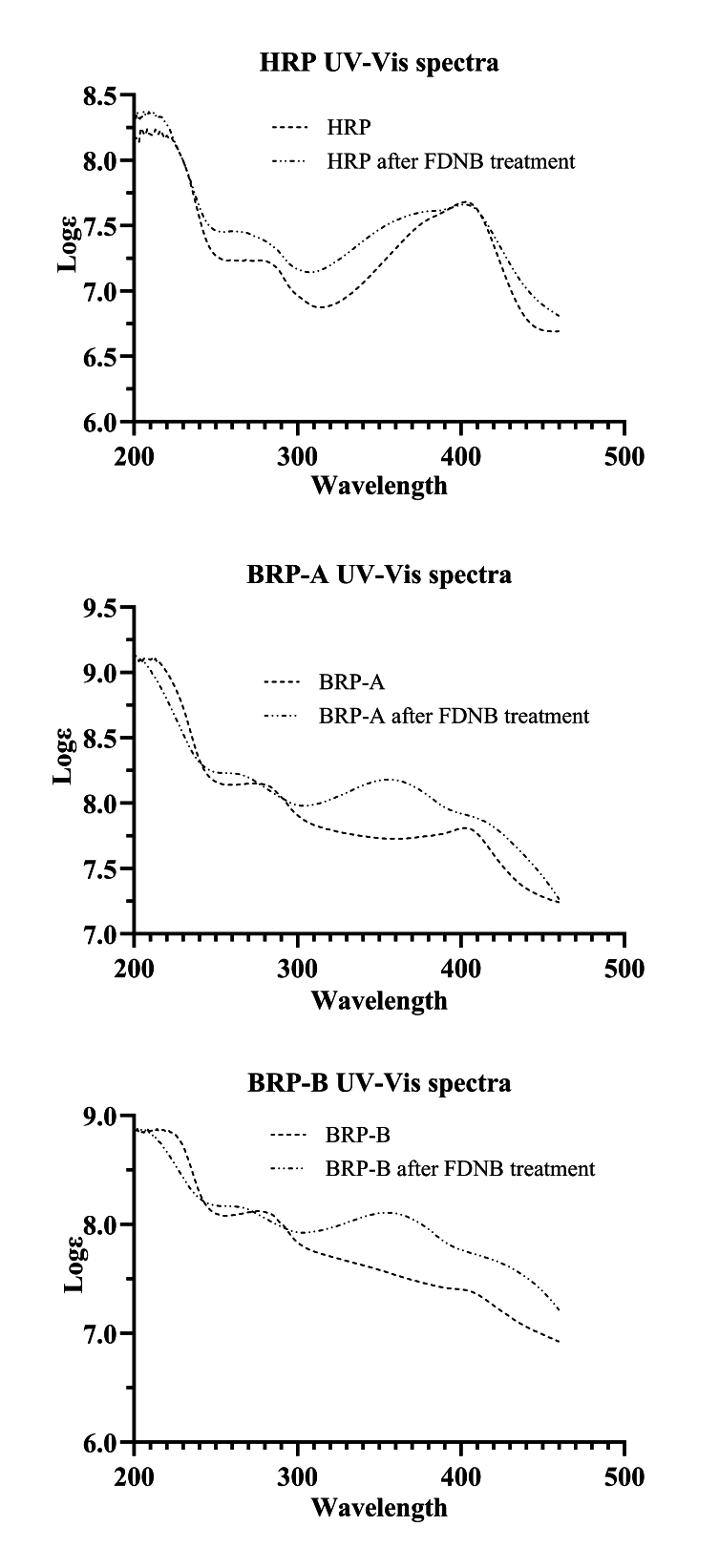
Supplementary Fig. S5. Comparison of the UV–Vis spectra for HRP, BRP-A, and BRP-B after treatment with FDNB.

**Figure:**
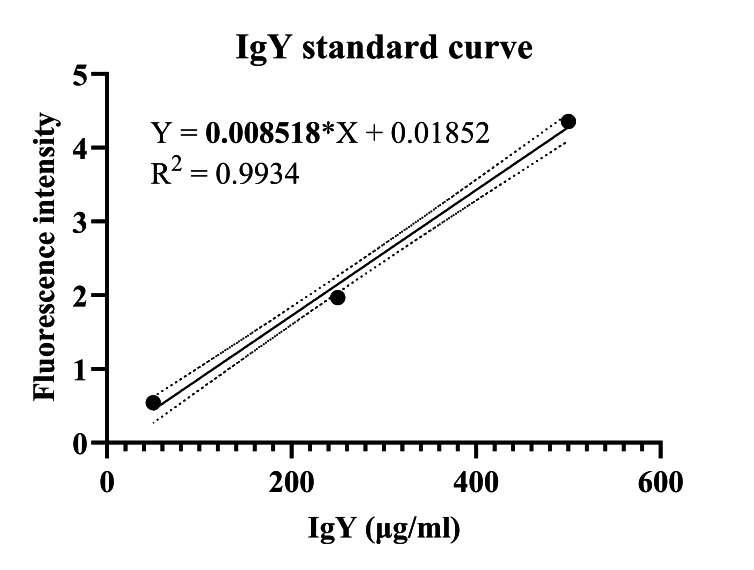
Supplementary Fig. S6. The standard curve generated using the fluoresce intensity obtained using various concentrations of IgY by the OPA method. The equation was used to determine the amine content of each conjugate equivalent to the IgY polyclonal antibody concentration.

**Figure:**
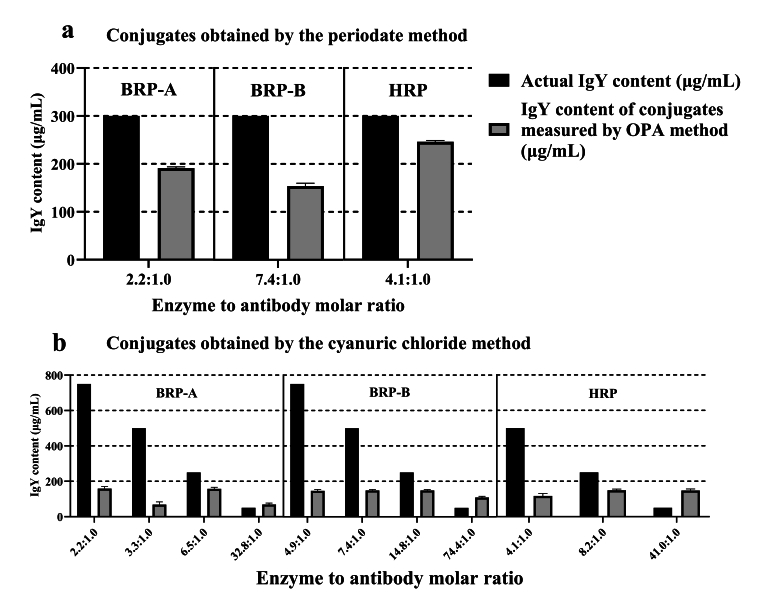
Supplementary Fig. S7. The estimation of the free amine content of the peroxidase-antibody conjugates obtained by the periodate (a) and cyanuric chloride method (b) using ortho-phthalaldehyde assay of free amine groups. IgY polyclonal antibody was used as standard for generating a standard curve, and the exact amount of IgY incorporated into each conjugate is shown (actual IgY content).
