# Supplementary material for: Application of two novel anionic peroxidases from Raphanus sativus L. var niger roots in labeling antibodies and developing an enzyme-linked immunosorbent assay
Source: Heliyon. 2024 Dec 13;11(1):e40894. doi: 10.1016/j.heliyon.2024.e40894 (PMC11720944; doi:10.1016/j.heliyon.2024.e40894)
Supplement: Multimedia component 1 [file mmc1.docx]

**Supplementary Table S1.** A brief list of HRP chromogenic substrates

| Substrate |  | Type of substrate |  | λ | ԑ (extinction coefficient)(product) |  | Solubility (in water, at RT) |  | Consideration | Ref. |
| --- | --- | --- | --- | --- | --- | --- | --- | --- | --- | --- |
| **4AA** (4-aminoantipyrine) |  | Soluble |  | 510 nm | 6.58 L･mmol^-1^･cm^-1^ |  | 50 mg/ml |  | Should be used with phenol as co-substrate. | (1, 2) |
| **ABTS** (2,2'-azino-bis(3-ethylbenzothiazoline-6-sulfonic acid) |  | Soluble |  | 405 nm | 36.8 L･mmol^-1^･cm^-1^ |  | 50 mg/mL |  | - | (3-5) |
| **o-Dianisidine** |  | Soluble |  | 460 nm | 30.0 L･mmol^-1^･cm^-1^ |  | 25 mg/ml |  | The measurement is performed at 560 nm in end-point assays. | (5-8) |
| **Guaiacol** |  | Soluble |  | 470 nm | 26.6 L･mmol^-1^･cm^-1^ |  | 24.8 mg/ml |  | - | (5, 9) |
| **Pyrogallol** |  | Soluble |  | 420 nm | 12.8 L･mmol^-1^･cm^-1^ |  | 380 mg/ml |  | - | (10, 11) |
| **OPD** (o-phenylenediamine) |  | Soluble |  | 417 nm | 16.7 L･mmol^-1^･cm^-1^ |  | 30 mg/ml |  | The oxidized form of the product has a λ_max_ of 490 nm. | (12, 13) |
| **TMB** (3,3',5,5'-tetramethylbenzidine) |  | Soluble/Precipitating |  | 652 nm | 39 L･mmol^-1^･cm^-1^ |  | 0.1 mg/ml |  | The oxidized form of the product has a λ_max_ of 450 nm. | (5, 14, 15) |
| **DAB** (3,3'-diaminobenzidine) |  | Precipitating |  | 480 nm | 5.5 L･mmol^-1^･cm^-1^ |  | 10 mg/ml |  | Limited application in quantitative assays. | (16-18) |
| **AEC** (3-amino-9-ethylcarbazole) |  | Precipitating |  | N/A | N/A |  | 0.1 mg/ml |  | Only for in vitro visualization purposes. | (19) |
| **4CN** (4-Chloro-1-Naphthol) |  | Precipitating |  | N/A | N/A |  | 0.1 mg/ml |  | Only for in vitro visualization purposes. Inferior as compared to other precipitating substrates. | (20) |

References:

1. Rosini E, Caldinelli L, Piubelli L. Assays of D-Amino Acid Oxidase Activity. Frontiers in Molecular Biosciences. 2018;4.

2. Sigma-Aldrich. Product specification sheet for 4-aminoantipyrine (CAS:83-07-8).

3. Sigma-Aldrich. Enzymatic Assay of Peroxidase (EC 1.11.1.7) 2,2'-Azino-bis(3-Ethylbenzthiazoline-6-Sulfonic Acid) as a Substrate [Available from: <https://www.sigmaaldrich.com/SE/en/technical-documents/protocol/protein-biology/enzyme-activity-assays/enzymatic-assay-of-peroxidase-abts-as-substrate>.

4. Sigma-Aldrich. Product information sheet for 2,2′-Azino-bis(3-ethylbenzthiazoline-6-sulfonic acid) (CAS: 30931-67-0).

5. Mika A, Luthje S. Properties of guaiacol peroxidase activities isolated from corn root plasma membranes. Plant Physiology. 2003;132(3):1489-98.

6. Sigma-Aldrich. Enzymatic Assay of Diamine Oxidase (E.C. No. 1.4.3.22) [Available from: <https://www.sigmaaldrich.com/SE/en/technical-documents/protocol/protein-biology/enzyme-activity-assays/enzymatic-assay-of-diamine-oxidase?srsltid=AfmBOoo3n9ubMyMVWaj2lp4l-69jG_YyqsemlgBuV9J8yOljsQAiTUoS>.

7. Dolz M, Monterrey DT, Beltrán-Nogal A, Menés-Rubio A, Keser M, González-Pérez D, et al. Chapter Four - The colors of peroxygenase activity: Colorimetric high-throughput screening assays for directed evolution. In: Renata H, editor. Methods in Enzymology. 693: Academic Press; 2023. p. 73-109.

8. Sigma-Aldrich. Product specification sheet for o-Dianisidine dihydrochloride (CAS: 20325-40-0).

9. Tam D, Varhanickova D, Shiu WY, Mackay D. Aqueous solubility of chloroguaiacols. Journal of Chemical and Engineering Data. 1994;39(1):83-6.

10. Schmidt TF, Caseli L, dos Santos DS, Oliveira ON. Enzyme activity of horseradish peroxidase immobilized in chitosan matrices in alternated layers. Materials Science and Engineering: C. 2009;29(6):1889-92.

11. Yalkowsky SH, He Y, Jain P. Handbook of aqueous solubility data: CRC press; 2016.

12. Fornera S, Walde P. Spectrophotometric quantification of horseradish peroxidase with o-phenylenediamine. Analytical biochemistry. 2010;407(2):293-5.

13. Yao G, Yao Q, Xia Z, Li Z. Solubility determination and correlation for o-phenylenediamine in (methanol, ethanol, acetonitrile and water) and their binary solvents from T=(283.15–318.15) K. The Journal of Chemical Thermodynamics. 2017;105:179-86.

14. Frey A, Meckelein B, Externest D, Schmidt MA. A stable and highly sensitive 3,3′,5,5′-tetramethylbenzidine-based substrate reagent for enzyme-linked immunosorbent assays. Journal of Immunological Methods. 2000;233(1):47-56.

15. Liem HH, Cardenas F, Tavassoli M, Poh-Fitzpatrick MB, Muller-Eberhard U. Quantitative determination of hemoglobin and cytochemical staining for peroxidase using 3,3′,5,5′-tetramethylbenzidine dihydrochloride, a safe substitute for benzidine. Analytical Biochemistry. 1979;98(2):388-93.

16. van Noorden CJ, Jonges GN. Molecular extinction coefficients of lead sulfide and polymerized diaminobenzidine as final reaction products of histochemical phosphatase reactions. Cytometry. 1992;13(6):644-8.

17. Sigma-Aldrich. Product information sheet for 3,3 Diaminobenzidine tetrahydrochloride hydrate (CAS: 868272-85-9).

18. Khramtsov P, Minin A, Galaeva Z, Mukhlynina E, Kropaneva M, Rayev M. Optimizing the Composition of the Substrate Enhances the Performance of Peroxidase-like Nanozymes in Colorimetric Assays: A Case Study of Prussian Blue and 3,3'-Diaminobenzidine. Molecules. 2023;28(22).

19. Sigma-Aldrich. Product information sheet for 3-Amino-9-ethylcarbazole (CAS: 132-32-1).

20. Safety data sheet for 4-Chloro-1-naphthol (CAS: 604-44-4). In: Roth C, editor.
