# Supplementary material for: Application of two novel anionic peroxidases from Raphanus sativus L. var niger roots in labeling antibodies and developing an enzyme-linked immunosorbent assay
Source: Heliyon. 2024 Dec 13;11(1):e40894. doi: 10.1016/j.heliyon.2024.e40894 (PMC11720944; doi:10.1016/j.heliyon.2024.e40894)
Supplement: Multimedia component 2 [file mmc2.docx]

| **Parameter** |  | **BRP-A** | |  | **BRP-B** |
| --- | --- | --- | --- | --- | --- |
| **K_m_, H_2_O_2_** (mM) |  | **0.043** |  | | **0.067** |
| **K_m_, 4AA** (mM) |  | **10.63** |  | | **15.38** |
| **Optimum pH** |  | **5** |  | | **6** |
| **V_max_** (U)**^*^** |  | **5.18** |  | | **2.88** |

**Supplementary Table S2.** An overview of the kinetic parameters of BRP-A and BRP-B

All the analyses were done using phenol and 4-aminoantipyrine as substrate.

*Extrapolated from the Lineweaver-Burk plot which was used to determine K_m_ for 4-aminoantipyrine. Depends on the amount of enzyme incorporated into the reaction mixture.
