# Supplementary material for: Application of two novel anionic peroxidases from Raphanus sativus L. var niger roots in labeling antibodies and developing an enzyme-linked immunosorbent assay
Source: Heliyon. 2024 Dec 13;11(1):e40894. doi: 10.1016/j.heliyon.2024.e40894 (PMC11720944; doi:10.1016/j.heliyon.2024.e40894)
Supplement: Multimedia component 3 [file mmc3.docx]

**Supplementary Table S8.** Calculation of limit of detection for HRP, BRP-A, and BRP-B conjugates prepared by cyanuric chloride and periodate method

| **Conjugation method** | **Type of peroxidase** | **Enzyme to antibody molar ratio** | **Limit of detection (ng per well)** | **Ab conc. (μg/mL)** | **Slope of standard curve** | **SD of blanks** | **R^2^** |
| --- | --- | --- | --- | --- | --- | --- | --- |
| **Cyanuric chloride** | **HRP** | **41.0** | **64.02** | **40** | **0.00112** | **0.0239** | **97.50** |
|  |  | **8.2** | **60.82** | **64** | **0.00146** | **0.0296** | **98.49** |
|  |  | **4.1** | **106.21** | **64** | **0.00087** | **0.0308** | **95.34** |
|  | **BRP-A** | **32.8** | **Values could not be established due to an extremely low signal-to-noise ratio** | | | | |
|  |  | **6.5** |  |  |  |  |  |
|  |  | **3.3** |  |  |  |  |  |
|  |  | **2.2** |  |  |  |  |  |
|  | **BRP-B** | **74.4** |  |  |  |  |  |
|  |  | **14.8** |  |  |  |  |  |
|  |  | **7.4** |  |  |  |  |  |
|  |  | **4.9** |  |  |  |  |  |
| **Periodate** | **HRP** | **4.1** | **43.60** | **10** | **0.00172** | **0.0250** | **97.36** |
|  | **BRP-A** | **3.3** | **385.71** | **64** | **0.00028** | **0.0360** | **99.83** |
|  | **BRP-B** | **7.4** | **213.75** | **64** | **0.00056** | **0.0399** | **99.30** |
